# A Reduction in Selenoprotein S Amplifies the Inflammatory Profile of Fast-Twitch Skeletal Muscle in the *mdx* Dystrophic Mouse

**DOI:** 10.1155/2017/7043429

**Published:** 2017-05-16

**Authors:** Craig Robert Wright, Giselle Larissa Allsopp, Alex Bernard Addinsall, Natasha Lee McRae, Sofianos Andrikopoulos, Nicole Stupka

**Affiliations:** ^1^Institute for Physical Activity and Nutrition Research (IPAN), School of Exercise and Nutrition Sciences, Deakin University, Geelong, VIC, Australia; ^2^Molecular Medical Research SRC, School of Medicine, Deakin University, Geelong, VIC, Australia; ^3^Department of Medicine, The University of Melbourne, Melbourne, VIC, Australia

## Abstract

Excessive inflammation is a hallmark of muscle myopathies, including Duchenne muscular dystrophy (DMD). There is interest in characterising novel genes that regulate inflammation due to their potential to modify disease progression. Gene polymorphisms in *Selenoprotein S* (*Seps1*) are associated with elevated proinflammatory cytokines, and in vitro SEPS1 is protective against inflammatory stress. Given that SEPS1 is highly expressed in skeletal muscle, we investigated whether the genetic reduction of *Seps1* exacerbated inflammation in the *mdx* mouse. F1 male *mdx* mice with a heterozygous *Seps1* deletion (*mdx*:*Seps1*^−/+^) were generated. The *mdx:Seps1*^−/+^ mice had a 50% reduction in SEPS1 protein expression in hindlimb muscles. In the extensor digitorum longus (EDL) muscles, mRNA expression of *monocyte chemoattractant protein 1* (*Mcp-1*) (*P* = 0.034), macrophage marker *F4/80* (*P* = 0.030), and *transforming growth factor-β1* (*Tgf-β1*) (*P* = 0.056) were increased in *mdx:Seps1*^−/+^ mice. This was associated with a reduction in muscle fibre size; however, ex vivo EDL muscle strength and endurance were unaltered. In dystrophic slow twitch soleus muscles, SEPS1 reduction had no effect on the inflammatory profile nor function. In conclusion, the genetic reduction of *Seps1* appears to specifically exacerbate the inflammatory profile of fast-twitch muscle fibres, which are typically more vulnerable to degeneration in dystrophy.

## 1. Introduction

Skeletal muscle repair is a highly coordinated process in which inflammation plays a vital role. Immediately following muscle damage, neutrophils infiltrate the damaged area where they release cytokines to promote inflammation and reactive oxygen species break down cellular constituents [1]. Secondly, monocytes infiltrate the muscle, mature into macrophages, and engulf the cellular debris [1]. This is a highly organised process that prepares the damaged tissue for repair. Macrophages, like neutrophils, release cytokines that either contribute to (proinflammatory) or control inflammation (anti-inflammatory). The release of cytokines, such as interleukin 6 (IL-6) [2] and tumour necrosis factor alpha (TNF*α*) [3], stimulates satellite cell activation and commitment to myogenesis. If the damage is chronic, or if inflammation is excessive and persists, this repair process fails and with time leads to muscle tissue loss and fibrosis [4], as is seen in Duchenne muscular dystrophy (DMD).

DMD is a fatal hereditary disease that affects approximately 1 in 3500 live male births [5]. The majority of boys with DMD are wheelchair bound in their teens and succumb to the disease by their early thirties from respiratory or cardiac failure [6]. Due to mutations in the dystrophin gene, dystrophic muscles are vulnerable to contraction-induced injury. This leads to constant muscle damage and degeneration, with persistent infiltration of neutrophils and macrophages and high levels of inflammatory cytokines within the muscle microenvironment and in circulation. Specifically, elevated levels of the proinflammatory cytokines TGF-*β* [7–9], TNF*α* [10], MCP-1 [11], and IL-6 [12] have been observed in skeletal muscle biopsies and serum samples from patients with DMD. The *mdx* mouse is the dystrophic murine model of DMD, whose pathology is also characterised by increased inflammation. Similar to the human disease, TGF-*β* [13] and TNF*α* [14] levels are increased in *mdx* skeletal muscles and are associated with inflammatory cell infiltration [15]. An excessive and dysregulated inflammatory profile further exacerbates degeneration and impairs regeneration in both human DMD and the *mdx* mouse [16].

There is no cure for DMD, with glucocorticoids being the only treatment demonstrating clinical efficacy [17]. The mechanism of action of glucocorticoids is not fully understood; however, an attenuation of inflammation is thought to contribute to their effectiveness [18]. Glucocorticoids are not without serious side effects, causing excessive weight gain, behavioural abnormalities, and osteoporosis in boys with DMD [19]. Depending on the underlying disease pathology, glucocorticoids also alter protein metabolism [20] and inhibit anabolism in healthy young adults [21]. An abundance of dystrophy research is focused on therapeutic agents and gene targets that control skeletal muscle inflammation [22, 23]. Various preclinical studies in *mdx* mice have attempted to attenuate inflammation in order to improve muscle function [24, 25]. These include administration of an antibody against TNF*α* (Remicade) [14], depletion of macrophage populations [26], and inhibitory drugs targeting inflammatory-mediated intracellular pathways such as nuclear factor kappa-light-chain-enhancer of activated B cells (NF-*κ*B) [27]. Whilst many of these approaches have been successful in ameliorating the dystrophic pathology of *mdx* mice, their translation to human trials and clinical practice has been unsuccessful [28].

The natural history of DMD is quite heterogeneous, with significant interpatient variability in regard to disease progression and skeletal, respiratory, and cardiac muscle function, as well as responsiveness to treatment [29]. For example, polymorphisms in the glucocorticoid receptor increase long-term sensitivity to glucocorticoid treatment in DMD patients [28]. Similarly, polymorphisms in the osteopontin gene have also been identified as an important genetic modifier of disease severity in DMD [29], whilst polymorphisms in TGF-*β* receptor 2 are strong predictors of osteopontin mRNA levels, highlighting a link between osteopontin, inflammation, and TGF-*β* signalling in DMD [30]. As such, there is great interest in identifying and characterising the multigenic polymorphisms that modulate DMD disease progression or responsiveness to treatment. Identifying these gene polymorphisms could provide insights into the cellular mechanisms underlying the link between inflammation, muscle degeneration, and inadequate repair in DMD and may have implications for improved design and outcomes of DMD clinical trials [29].

Selenoprotein S (SEPS1) is an endoplasmic reticulum (ER) resident transmembrane selenoprotein with a C terminal selenocysteine residue that has antioxidant properties and is protective against ER stress [31, 32]. More than 15 polymorphisms in the *SEPS1* gene have been identified in humans, ranging in frequencies from approximately 1% to more than 32% [33]. The 105G → A polymorphism in the *SEPS1* promoter region has been particularly well characterised. It is thought to reduce SEPS1 expression and is associated with diseases characterised by heightened inflammation and oxidative stress [33]. In vitro studies demonstrate that reduced SEPS1 expression increases oxidative and ER stress in various mammalian cell lines [34–36]. In vitro, *Seps1* gene expression is increased by proinflammatory cytokines [37], perhaps as a protective strategy, since gene knockdown of *Seps1* increases proinflammatory cytokine mRNA levels in cultured RAW264.7 macrophages [31]. Using siRNA knockdown strategies in a mouse model of lipopolysaccharide-induced sepsis, He et al. [38] demonstrated a protective, anti-inflammatory effect of SEPS1 in vivo. Recently, selenoprotein N (*SepN1*) polymorphisms have been identified to cause congenital muscular dystrophy [39]. Given that SEPS1 is highly expressed in skeletal muscle [40] and is associated with inflammation, our aim was to investigate whether the genetic reduction of *Seps1* in *mdx* dystrophic mice would exacerbate skeletal muscle inflammation and compromise dystrophic hindlimb muscle structure and function. Therefore, we tested whether SEPS1 is a novel disease modifying gene in muscular dystrophy and myopathy.

## 2. Methods

### 2.1. Animals

C57BL/6 mice with a global *Seps1* heterozygous deletion were produced using the Cre/loxP system. The floxed *Seps1* gene was cleaved by a 3-phosphoglycerate kinase (PGK) promoter-driven Cre. These mice were subsequently crossed with female *mdx* mice on a C57BL/10 background to generate F1 male *mdx*:*Seps1*^−/+^ mice, with a genetic reduction of *Seps1*, and control *mdx*:*Seps1*^+/+^ littermates on a mixed C57BL/10 and C57BL/6 background. Mice were housed in standard laboratory conditions of temperature (22 ± 2°C) and relative humidity (55 ± 8%), with a 12-hour light/dark cycle. Animals had free access to water and were fed a standard chow diet. All animal experiments were conducted with the full approval of the Animal Ethics Committee Geelong, Deakin University (G29/2014). All procedures were conducted in accordance with the Australian Code of Practice for the Care and Use of Animals for Scientific Purposes.

### 2.2. Body Composition and Whole Body Metabolism

Between six and 12 weeks of age, body composition was determined by magnetic resonance imaging (MRI) (Body Composition Analyser ESF-005, EchoMRI™). At 11 weeks of age, oxygen consumption (VO₂; ml kg min), carbon dioxide production (VCO₂; ml kg min), and respiratory exchange ratio (RER) were measured over a 24-hour period using a metabolic analyser (Accuscan Fusion v3.6; Columbus Instruments International). During this 24-hour period, total and ambulatory movement was assessed with an Animal Activity Meter (Opto-Varimex-Mini; Columbus Instruments International).

### 2.3. Ex Vivo Analysis of Muscle Function

At 12 weeks of age, mice were anaesthetised with an intraperitoneal injection of medetomidine (0.6 mg/kg), midazolam (5 mg/kg), and fentanyl (0.05 mg/kg), such that they were unresponsive to tactile stimuli. EDL and soleus muscles were dissected from the proximal to distal tendon, and each tendon was tied off with 4-0 surgical grade sutures. Excised muscles were transferred to the 1300A Whole Mouse Test System organ bath (Aurora Scientific), submerged in Krebs Ringer solution (137 mM NaCl, 24 nM NaHCO_3_, 11 mM D-glucose, 5 mM KCl, 2 mM CaCl_2_, 1 mM NaH_2_PO_4_H20, 1 mM MgSO_4_, and 0.025 mM d-tubocurarine chloride), continuously perfused with carbogen (5% CO_2_ in O_2_), and thermostatically maintained at 25°C. The distal tendon was attached to an immobile pin and the proximal tendon to the lever arm of a dual mode force transducer (300-CLR; Aurora Scientific). EDL and soleus muscles were stimulated by supramaximal square wave pulses, for a duration of 350 msec and 1200 msec, respectively. Stimulation procedures and contractile responses were controlled and measured using Dynamic Muscle Control computer software (DMCv5.415), with on board controller interfaced with transducer control/feedback hardware (Aurora Scientific) [41].

All protocols were performed at optimal muscle length (*L*_O_). Maximum isometric tetanic force (*P*_O_) production was established from the plateau in the force frequency curve: 10, 30, 50, 60, 80, 100, and 120 Hz for EDL and 10, 20, 30, 50, 60, 80, 100, and 120 Hz for soleus. For determination of fatigability, muscles underwent a four-minute submaximal protocol, consisting of 60 Hz stimulations, once every five seconds. Recovery from fatigue was determined by 60 Hz stimulations, administered at two, five, and 10 minutes following the fatigue protocol [41, 42].

Optimum fibre length (*L*_f_) was calculated by multiplying *L*_O_ by predetermined *L*_f_/*L*_O_ ratios, 0.44 for EDL and 0.71 for soleus [43, 44]. Cross-sectional area of muscle samples was then determined by dividing muscle mass (mg) by the product of *L*_f_ and 1.06 mg.mm^3^, the density of mammalian muscle [43]. *P*_O_ values were normalised to muscle cross-sectional area and expressed as specific force (sP_O_), to account for variation in muscle size.

Following muscle function testing, EDL, soleus, and tibialis anterior (TA) muscles were trimmed of tendons, weighed, and snap frozen in liquid nitrogen. EDL and soleus muscles excised from the contralateral hindlimb were embedded in optimal cutting temperature compound (TissueTek OCT Compound) and frozen in thawing isopentane for histological analysis.

### 2.4. Western Blotting to Confirm the Genetic Reduction of Seps1

Approximately, 20 mg of TA muscle was homogenised in 1x radioimmunoprecipitation assay (RIPA) lysis buffer containing 1x protease inhibitor cocktail (Sigma-Aldrich) and quantified by the BCA Protein Assay kit (Thermo Scientific). Alternatively, 10 *μ*m thick EDL muscle cross sections were placed into 50 *μ*l of 2x Laemmli sample buffer and underwent two freeze thaw cycles at −80°C. Proteins (7.5 *μ*g of unfractionated TA homogenates or 15 *μ*l of EDL cryosection lysates) were separated on an Any kD™ Mini-PROTEAN® TGX Stain-Free™ gel (BioRad). Gels were then activated and imaged using the ChemiDoc MP System (BioRad) as per manufacturer's instructions and then transferred onto a PVDF membrane. These were blocked with 5% skim milk/TBST, incubated with an anti:SEPS1 antibody (HPA010025; Sigma-Aldrich; diluted 1 : 200 in 1% skim milk/TBST), and followed by a goat anti-rabbit horse radish peroxidase (HRP) secondary antibody (Cell Signaling Technologies: diluted 1 : 5000). SEPS1 bands were detected with enhanced chemiluminescence and imaged using the ChemiDoc MP System. The optical density of the SEPS1 bands and the protein bands on the TGX Stain-Free gels were analysed using Image Lab™ software (Bio-Rad), and SEPS1 protein levels were normalised to the total optical density of all protein bands.

### 2.5. Real Time Quantitative PCR (qPCR) to Characterise the Inflammatory Profile

As previously described [45], whole soleus and EDL muscles were manually homogenised in Tri-Reagent® solution (Ambion Inc.). Total cellular RNA was extracted and purified using an RNeasy® Mini Kit (Qiagen), and 1 *μ*g was reverse transcribed using a High Capacity cDNA Reverse Transcription kit (Applied Biosystems, Warrington, UK). Gene expression was measured using Power SYBR® Green PCR Master Mix (Applied Biosystems) and 300 nM primers (See Supplementary Table 1 available online at https://doi.org/10.1155/2017/7043429). Results were normalised to the housekeeping gene glyceraldehyde-3-phosphate dehydrogenase (*Gapdh*) and verified with *β-actin*, which was not significantly different to the *Gapdh* results [46].

### 2.6. Histology and Immunohistochemistry

Eight *μ*m of EDL muscles were transversely sectioned using a cryostat maintained at −20°C (CM1860 Cryostat, Leica, North Ryde, AUS) and placed on silane-coated slides (Star Frost microscope slides, ProSciTech). Sections were stained with Mayer's haematoxylin solution (Sigma-Aldrich) and Eosin (Sigma-Aldrich) and then imaged with a digital camera mounted on a DM1000 microscope (Leica) at 100x magnification. To determine fibre size, the minimum Feret's diameter was measured [47] using Image-Pro Plus (Media Cybernetics, Rockville, USA) (*n* = 10; >500 fibres per muscle). The percentage of muscle fibres with centrally located nuclei was also assessed (*n* = 6; >200 fibres per muscle).

To assess SEPS1 immunoreactivity, EDL muscle cross sections were fixed in 4% paraformaldehyde (PFA) and permeabilised in tris buffered saline (TBS) containing 0.5% Triton X-100. Sections were probed with the anti-SEPS1 antibody (1 : 200 dilution) and followed by Alexa Fluor 594 goat anti-rabbit 2° antibody (A11012; Invitrogen; 1 : 1500 dilution). Nuclei were counter stained with DAPI containing mounting medium (Vectashield).

### 2.7. Statistical Analysis

All results are presented as mean ± standard error of the mean (SEM). Two-way repeated measures ANOVA with Šídák post hoc analysis and unpaired Student's *t*-test were performed using Prism 6 (GraphPad, San Diego, CA), with a *P* < 0.05 used for significance.

## 3. Results

### 3.1. Confirmation of the Genetic Reduction of Seps1

**Figure 1:**
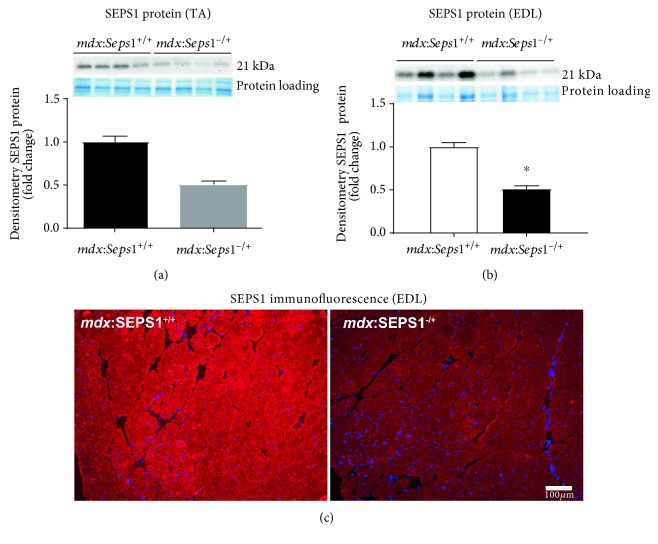
SEPS1 protein expression in fast-twitch hindlimb muscles. Protein expression of the 21 kDa SEPS1 protein in (a) TA and (b) EDL muscles using TGX Stain-Free gel technology to control for even protein loading (*n* = 8 and 4, resp.). ^∗^*P* < 0.001 using Student's *t*-test. (c) Representative SEPS1 immunoreactivity in transverse EDL muscle cross sections shown SEPS1 expression by all skeletal muscle fibres (*n* = 3).

There was an approximate 50% reduction in SEPS1 protein expression in TA and EDL hindlimb muscles from *mdx*:*Seps1*^−/+^ mice compared to *mdx:Seps1^+/+^* littermates (Figures 1(a) and 1(b), resp.). Using immunofluorescent staining, SEPS1 expression was confirmed in all EDL muscle fibres (Figure 1(c)).

### 3.2. Body Composition

**Figure 2:**
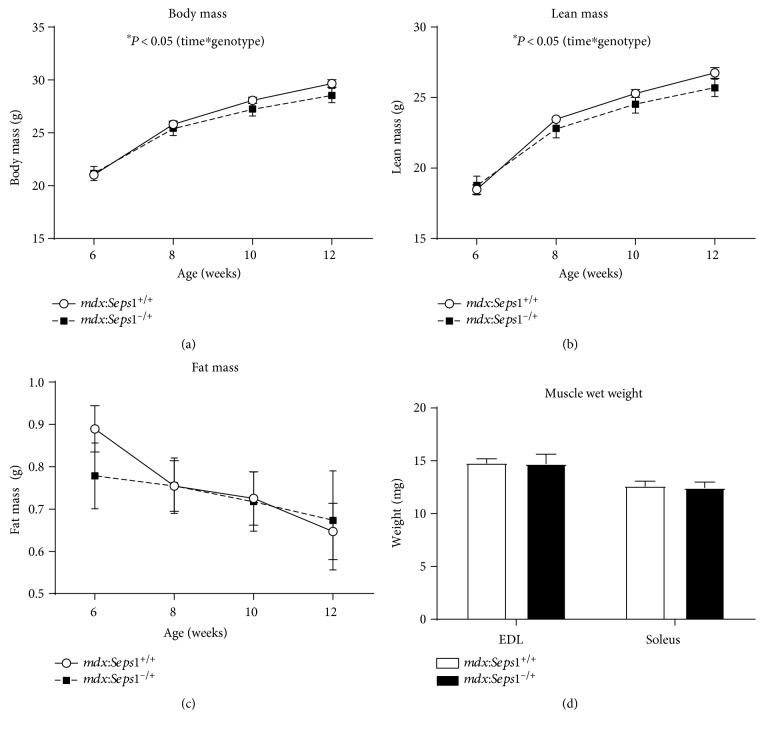
Body composition between 6 and 12 weeks of age. (a) Body mass, (b) lean mass and (c) fat mass between 6 and 12 weeks of age (*n* = 13), and (d) muscle wet weight of isolated EDL and soleus muscles (*n* = 11). ∗ indicates significant interaction (time^∗^genotype) using 2-way repeated measures ANOVA (*P* < 0.05).

Body composition was measured fortnightly between six and 12 weeks of age. Over the six-week measurement period, *mdx*:*Seps1*^−/+^ mice had smaller gains in total body mass (Figure 2(a)) and lean mass (Figure 2(b)) compared to those of the *mdx* littermates using a 2-way repeated measures ANOVA (time∗genotype interaction, *P* = 0.037 and *P* = 0.013, resp.). Typical of the dystrophic phenotype [48], both genotypes demonstrated significant and similar losses in fat mass (Figure 2(c)) between six and 12 weeks. The wet weight of excised EDL and soleus muscles was measured and was not significantly different between groups (Figure 2(d)).

### 3.3. Metabolic Profile

**Figure 3:**
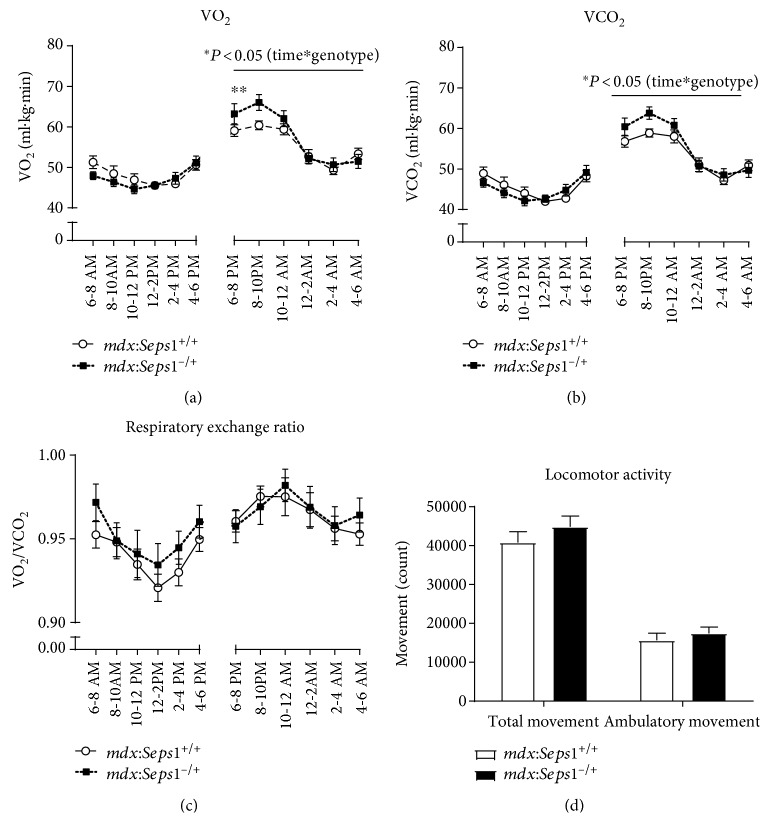
Whole body metabolism and spontaneous physical activity. (a) Oxygen consumption (VO₂), (b) carbon dioxide production (VCO₂), (c) respiratory exchange ratio (RER), and (d) locomotor activity at 11 to 12 weeks of age (*n* = 13). ^∗∗^Significant Šídák post hoc analysis (*P* < 0.05).

Whole body metabolism and spontaneous physical activity of the mice were assessed using metabolic cages and animal activity meters at 11 weeks of age [48]. Both genotypes had higher VO₂, VCO₂, and RER values between 6 pm and 6 am (night) during their active period, compared to 6 am–6 pm (Figure 3(a) and 3(b)). No differences in VO₂, VCO₂, or RER were detected between genotypes during the day, and no differences in RER were detected between genotypes at night (Figure 3(c)). However, a 2-way repeated measures ANOVA revealed that the *mdx*:*Seps1*^−/+^ mice had higher VO₂ (*P* < 0.05) and VCO_2_ (*P* < 0.05) values between 6 pm and 6 am. Post hoc analysis revealed that the *mdx*:*Seps1*^−/+^ mice had a higher VO₂ during the first two active hours at night (Figure 3(a)).

Analysis of total and ambulatory movement over the same 24-hour period revealed no differences between genotypes (Figure 3(d)).

### 3.4. Ex Vivo Skeletal Muscle Function

**Figure 4:**
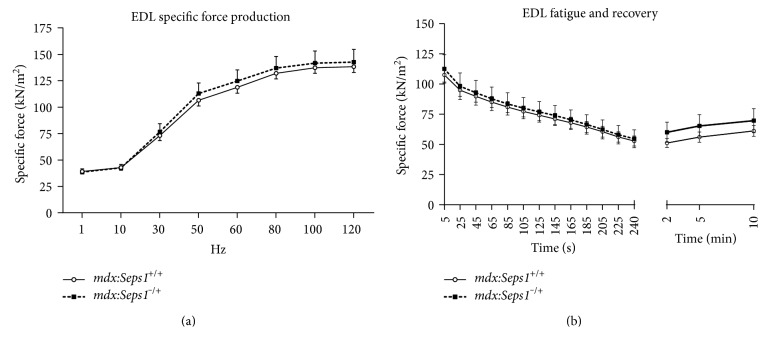
Ex vivo strength and fatigue analysis of the *EDL*. (a) Force frequency curve of the *EDL* muscle in response to ex vivo stimulation to assess muscle strength normalised to muscle size (specific force; sP_O_) and (b) sP_O_ of the *EDL* during four minutes of intermittent submaximal fatiguing stimulation and force recovery at 2, 5, and 10 minutes (*n* = 11).

The EDL and soleus muscles had no significant differences in specific force (sP_O_) production, fatigability, and recovery from fatigue between the *mdx*:*Seps1*^−/+^ mice and *mdx* littermate controls (Figure 4 and Supplementary Figure 1, resp.). This indicates that at 12 weeks of age, the genetic reduction of *Seps1* has no effect on dystrophic hindlimb muscle function.

### 3.5. Inflammatory Profile of *mdx* Skeletal Muscle

**Figure 5:**
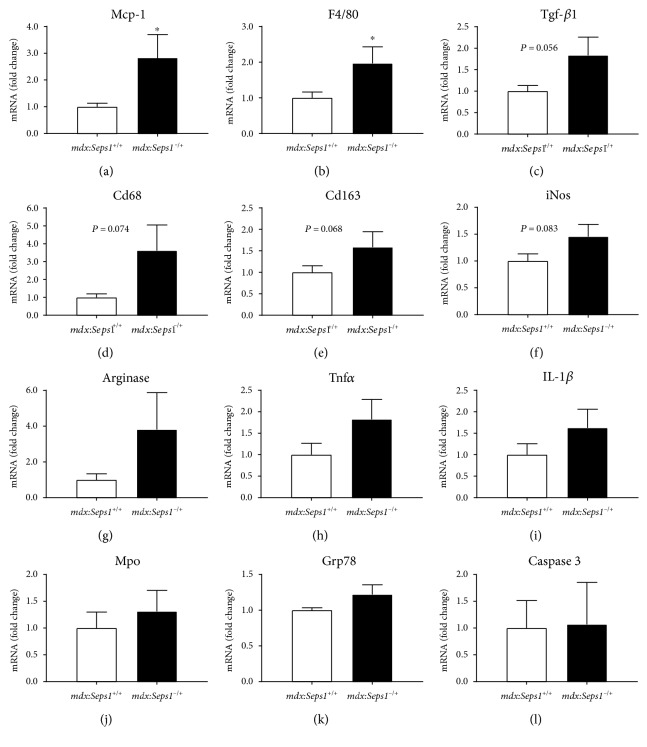
Inflammatory, ER stress, and cell death gene markers in *EDL* muscles. (a) Monocyte chemoattractant protein 1 *(Mcp-1),* (b) EGF-like module-containing mucin-like hormone receptor-like 1 (*F4/80*), (c) transforming growth factor *β*1 (*Tgf-β1*), (d) cluster of differentiation 68 (*Cd68*), (e) cluster of differentiation 163 (*Cd163*), (f) inducible nitric oxide (*iNos*), (g) arginase, (h) tumour necrosis factor *α* (*Tnfα*), (i) interleukin 1*β* (*IL-1β*), (j) *myeloperoxidase* (*Mpo*), (k) glucose-regulated protein 78 (*Grp78*), and (l) caspase 3 gene expression in the *EDL* muscle at 12 weeks of age, represented as fold change ± SEM (*n* = 11). ^∗^*P* < 0.05 using Student's *t*-test.

To investigate the inflammatory profile of *mdx* skeletal muscles following the genetic reduction of *Seps1*, key inflammatory markers were examined using qPCR. In fast-twitch EDL muscles, *mdx:Seps1*^−/+^ mice had a 2.8-fold increase in mRNA expression of the inflammatory cytokine *Mcp-1* (*P* = 0.034) and a 2.0-fold increase in mRNA expression of the macrophage marker *F4/80* (*P* = 0.029) compared to *mdx* littermates (Figure 5(a) and 5(b)). A trend for increased mRNA transcript abundance was also observed for *Tgf-β1* (*P* = 0.056) and the macrophage markers *Cd68* (*P* = 0.074) and *Cd163* (*P* = 0.068) in *EDL* muscles of *mdx*:*Seps1*^−/+^ mice (Figure 5(c), 5(d), and 5(e)). There was no significant difference in *iNos*, a marker for M1 proinflammatory macrophages, or *arginase* mRNA, a marker for M2 anti-inflammatory macrophages (Figure 5(f) and 5(g)), nor the *iNos/Arginase* ratio that is indicative of macrophage polarity towards an M1/M2 phenotype [49]. Similarly, there was no significant difference in *Tnfα* or *IL-1β* mRNA between genotypes (Figure 5(h) and 5(i)) nor in the neutrophil marker *myeloperoxidase* (*Mpo*) (Figure 5(j)). These data suggest that in fast-twitch muscles, SEPS1 might have a greater effect on macrophage infiltration, but not polarisation towards an anti- or proinflammatory phenotype.

Despite SEPS1 being associated with ER stress and apoptosis [35], the mRNA transcripts of the ER stress marker *glucose-regulated protein 78 (Grp78)* and the apoptosis marker *Caspase 3* (Figure 5(k) and 5(l)) were similar in *mdx*:*Seps1*^−/+^ mice and *mdx* littermates.

Whilst the genetic reduction of Seps1 resulted in moderate changes in the inflammatory profile of the dystrophic fast-twitch EDL muscle, the inflammatory profile of the slow-twitch soleus muscle was not changed (Supplementary Figure 2).

### 3.6. Skeletal Muscle Morphology of the EDL

**Figure 6:**
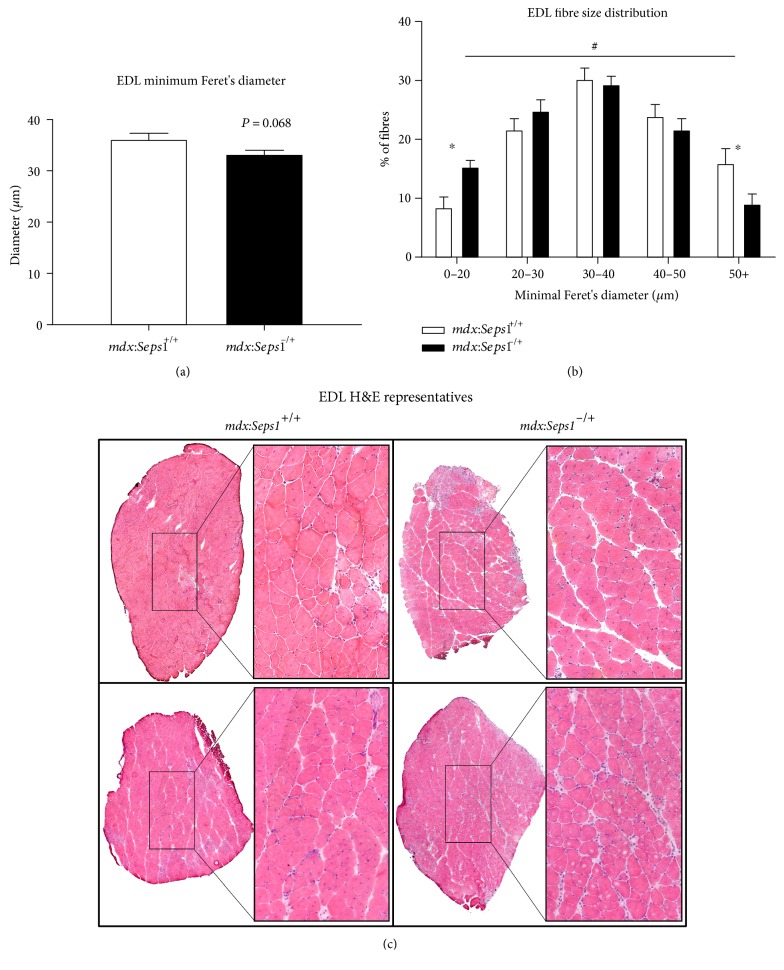
Histological analysis of EDL muscle. (a) Minimum Feret's diameter, (b) fibre size distribution, and (c) representative transverse H&E sections of the EDL muscle at 12 weeks of age (*n* = 10). ^#^*P* < 0.05 two-way ANOVA, ^∗^*P* < 0.05 Šidák post hoc.

Due to the heightened inflammatory state of EDL muscles and the smaller gain of lean mass observed in *mdx*:*Seps1*^−/+^ mice compared to *mdx* littermates, morphological analysis of H&E stained EDL cross sections was performed. A trend for smaller muscle fibres was observed in *mdx*:*Seps1*^−/+^ mice compared to *mdx* littermates (*P* = 0.068) (Figure 6(a)). When results were further categorised according to cross-sectional area [50], 2-way repeated measures ANOVA and post hoc analysis revealed that *mdx*:*Seps1*^−/+^ mice had significantly more small fibres (0–20 *μ*M min Ferets diameter (*P* < 0.05) and fewer large fibres (>50 *μ*m min Ferets diameter; (*P* < 0.05)) compared to *mdx* littermates (Figure 6(b)). To assess whether this increase in small muscle fibres in *mdx*:*Seps1*^−/+^ mice was due to increased regeneration, the proportion of EDL fibres with centrally located nuclei, a hallmark of a recently repaired muscle fibres [51], was assessed. However, no differences were found, with approximately 75% of fibres possessing central nuclei across both genotypes (data not shown).

## 4. Discussion

Here, we identified SEPS1 as a potential modifier of skeletal muscle inflammation using a murine model of DMD, the *mdx* mouse. For the first time, this study has shown that reduced SEPS1 expression, via heterozygous global deletion of *Seps1*, exacerbates the inflammatory profile in the fast-twitch EDL muscle, but not the slower twitch soleus muscle. This was associated with a reduction in total body mass and lean mass gain between six and 12 weeks of age, a shift in fibre size distribution towards a smaller cross-sectional area, and an increase in whole body VO₂ consumption and VCO_2_ production. Despite these changes in growth, muscle fibre size, inflammation, and metabolism, there were no changes in strength and endurance of hindlimb muscles at 12 weeks of age.

In many disease states including muscular dystrophy, fast-twitch fibres are lost first [52, 53]. Importantly, this study shows that a genetic deletion of *Seps1* leads to an increase in muscle inflammation of the fast-twitch EDL muscles in the *mdx* mouse. Here, mRNA transcripts of *Mcp-1*, a chemokine that attracts monocytes to the damaged area, were 2.8-fold higher, and the macrophage marker *F4/80* mRNA was 2-fold higher in the *mdx:*Seps1^−/+^ EDL muscle compared to *mdx* littermates. There was a trend for increased gene expression of *Tgf-β1* and macrophage markers *Cd68* and *Cd163* with the genetic reduction of *Seps1* in dystrophic EDL muscles. Previous ex vivo studies on macrophages and macrophage cell lines (RAW264.7) have consistently found exacerbation of the inflammatory profile upon SEPS1 knockdown [31, 54, 55]. This is interesting as it was macrophage markers and *Mcp-1* mRNA levels that were elevated in our study. Given that fast-twitch fibres are typically more susceptible to ER, oxidative, and inflammatory stress [56], there could be a greater need for the protective effects of SEPS1, where excessive macrophage infiltration exacerbates degeneration and fibrosis. Interestingly, these changes in inflammatory gene expression did not occur in dystrophic soleus muscles following the genetic reduction of *Seps1*.

Fast-twitch muscles are generally more susceptible to contraction-induced injury [57]. The *mdx* mouse hindlimb muscles are predominantly fast-twitch with the exception of the soleus that has approximately 60% slow-twitch type-1 muscle [58], which could explain the lack of increased inflammation with the genetic reduction of *Seps1* in *mdx* soleus muscles (Supplementary Data). MCP-1 is a potent chemotactic factor that recruits and activates monocyte cells at the site of damage [5, 59], and this may explain why *Mcp-1* and the macrophage cell surface marker *F4/80* were elevated simultaneously. Though other inflammatory markers measured were not significantly different between groups, all trended in the same positive direction, suggesting that the inflammatory profile of dystrophic EDL muscles is exacerbated by the genetic reduction of *Seps1*. Whilst highly expressed in skeletal muscle fibres, SEPS1 is ubiquitously expressed. Given the global knockdown model used, reduction of SEPS1 in infiltrating inflammatory cells and fibroblasts may have also contributed to the exacerbated inflammatory profile observed in EDL muscles of *mdx*:Seps1^−/+^ mice. Given chronic inflammation reduces protein synthesis and can cause muscle atrophy [4], the reduced muscle fibre size in the *mdx:Seps1*^−/+^ mice is potentially explained by the increased inflammatory profile in these muscles.

In *mdx* skeletal muscle, *Mcp-1* mRNA is already 15–36-fold higher and macrophage infiltration is similarly elevated [5]. *Tgf-β1* mRNA is also chronically elevated in *mdx* mice and is associated with impaired regeneration, atrophy, and fibrosis [60]. Therefore, this model may somewhat mask the effect of reduced SEPS1 in skeletal muscle, and an acute trauma to skeletal muscle may elucidate a more clear role for SEPS1 in skeletal muscle. To better elucidate the role of SEPS1 in dystrophic muscles, the pathology of *mdx* mice should be exacerbated in follow-up studies using acute and chronic exercise to increase muscle damage and inflammation [61]. The role of SEPS1 in inflammation in otherwise healthy, nondystrophic muscles following an acute injury should also be investigated. In dystrophic muscles, injury and inflammation occur in a very different cellular milieu than acute injury and repair in nondystrophic muscles. These differences are due to the chronic contraction-induced injury occurring in dystrophic muscles and have been described as “smoldering inflammation” [59] and as “asynchronously regenerating microenvironments” [16]. This has implications for muscle inflammation and effective repair, as different pathways may need to be targeted in dystrophic compared to nondystrophic muscles. For example, in *mdx* mice, inhibition of MCP-1 signalling via the CCR2 receptor ameliorated inflammation and improved the pathology dystrophic muscles [5]. Whereas, in normal muscles, following acute injury inhibition of CCR2 signalling reduces macrophage accumulation and impedes successful regeneration [62]. This is interesting, as we observed increased mRNA transcripts of *Mcp-1*, a CCR2 ligand, and the macrophage marker *F4-80* with the genetic reduction of *Seps1*.

Heightened inflammation and inflammatory pathologies, including muscular dystrophy, are associated with higher metabolic costs [63, 64]. Importantly, tissue metabolism can be upregulated by increased infiltration of inflammatory cells that can increase their oxygen demand by 50-fold through the generation of ROS [63, 65]. In our study, reduced SEPS1 expression increased inflammation, and this was associated with increases in O_2_ consumption and CO_2_ production, without shifting substrate utilisation (as estimated by RER). This suggests that the SEPS1 reduction may exacerbate muscle inflammation, driving up tissue metabolism (for example, through the generation of ROS), and thus leading to a reduction in protein accretion. Despite the global knockout mouse model used, we would argue that effects on whole body metabolism are predominantly skeletal muscle specific. When compared to wild-type mice, *mdx* mice have increased whole body metabolism, including resting energy expenditure and protein turnover [48]. The hypothesis of a more severe pathology in our SEPS1 model is supported by the increased O_2_ consumption and CO_2_ production, smaller muscle fibres, and reduced lean mass gain in the *mdx*:*Seps1*^−/+^ mice.

Despite the changes in the inflammatory profile and muscle fibre size of the EDL muscles, at 12 weeks of age, the genetic reduction of *Seps1* did not impair *mdx* hindlimb muscle function ex vivo, nor did it reduce spontaneous physical activity. Although *mdx* mice have reduced spontaneous physical activity and show functional declines in muscle early in life, the *mdx* pathology is less severe than that of their human counterparts. Despite the ongoing degeneration, regeneration, and elevated inflammation [66], adult *mdx* mice may not display functional muscle impairment up to one year of age [61]. Therefore, differences in selenoprotein levels up to 12 weeks may have been insufficient to elucidate any perturbations in muscle function. A longer study duration should be considered to better elucidate the role of SEPS1 in the pathology of dystrophic *mdx* muscles.

## 5. Conclusions

The present study identified SEPS1 as a novel modulator of skeletal muscle inflammation in the fast-twitch muscles of *mdx* mice. Specifically, the genetic reduction of *Seps1* exacerbates inflammation in the fast-twitch EDL. Future research is needed to determine if SEPS1 affects disease progression in muscular dystrophy and whether SEPS1 could translate to other myopathies also significantly impacted by inflammation.

## Supplementary Material

TABLE 1: Mouse Primers Used for qPCR. Cluster of differentiation 68 (CD68), cluster of differentiation 163 (CD163), EGF-like module-containing mucin-like hormone receptor-like 1 (F4/80), glyceraldehyde 3-phosphate dehydrogenase (GAPDH), glucose-regulated protein 78 (Grp78), interleukin 1β (IL-1Β), inducible nitric oxide (iNOS), monocyte chemoattractant protein 1 (MCP-1), myeloperoxidase (MPO), transforming growth factor Β1 (TGF-Β1),tumour necrosis factor a (TNFa). SUPPLEMENTARY FIGURE 1: Ex vivo strength and fatigue analysis of the soleus. (a) Force Frequency curve of the soleus muscle in response to ex vivo stimulation, and (b) specific force production of the soleus during a 4 minute submaximal fatiguing stimulation, and force recovery at 2, 5 and 10 minutes (n⁼11). SUPPLEMENTARY FIGURE 2: Inflammatory gene profile of the soleus. (a) Monocyte chemoattractant protein 1 (MCP-1), (b) EGF-like module-containing mucin-like hormone receptor-like 1 (F4/80), (c) transforming growth factor β1 (TGF-Β1), (d) interleukin 6 (IL-6), (e) interleukin 1β (IL-1Β) and (f) tumour necrosis factor a (TNFa) gene expression in the soleus muscle at 12 weeks of age. Data are represented as fold change ± SEM, and are normalised to GAPDH. No differences in GAPDH expression were present between groups (n⁼11).
