# Supplementary material for: A Reduction in Selenoprotein S Amplifies the Inflammatory Profile of Fast-Twitch Skeletal Muscle in the mdx Dystrophic Mouse
Source: Mediators Inflamm. 2017 May 16;2017:7043429. doi: 10.1155/2017/7043429 (PMC5448157; doi:10.1155/2017/7043429)
Supplement: Supplementary file 1 — TABLE 1: Mouse Primers Used for qPCR. Cluster of differentiation 68 (CD68), cluster of differentiation 163 (CD163), EGF-like module-containing mucin-like hormone receptor-like 1 (F4/80), glyceraldehyde 3-phosphate dehydrogenase (GAPDH), glucose-regulated protein 78 (Grp78), interleukin 1β (IL-1Β), inducible nitric oxide (iNOS), monocyte chemoattractant protein 1 (MCP-1), myeloperoxidase (MPO), transforming growth factor Β1 (TGF-Β1),tumour necrosis factor a (TNFa). SUPPLEMENTARY FIGURE 1: Ex vivo strength and fatigue analysis of the soleus. (a) Force Frequency curve of the soleus muscle in response to ex vivo stimulation, and (b) specific force production of the soleus during a 4 minute submaximal fatiguing stimulation, and force recovery at 2, 5 and 10 minutes (n⁼11). SUPPLEMENTARY FIGURE 2: Inflammatory gene profile of the soleus. (a) Monocyte chemoattractant protein 1 (MCP-1), (b) EGF-like module-containing mucin-like hormone receptor-like 1 (F4/80), (c) transforming growth factor β1 (TGF-Β1), (d) interleukin 6 (IL-6), (e) interleukin 1β (IL-1Β) and (f) tumour necrosis factor a (TNFa) gene expression in the soleus muscle at 12 weeks of age. Data are represented as fold change ± SEM, and are normalised to GAPDH. No differences in GAPDH expression were present between groups (n⁼11). [file 7043429.f1.pdf]

SUPPLEMENTARY DATA

TABLE 1: Mouse Primers Used for qPCR. Cluster of differentiation 68 (CD68), cluster of differentiation 163 (CD163), EGF-like module-containing mucin-like hormone receptor-like 1 (F4/80), glyceraldehyde 3-phosphate dehydrogenase (GAPDH), glucose-regulated protein 78 (Grp78), interleukin 1β (IL-1β), inducible nitric oxide (iNOS), monocyte chemoattractant protein 1 (MCP-1), myeloperoxidase (MPO), transforming growth factor β1 (TGF-β1),tumour necrosis factor α (TNFα).

Gene	GenBank Accession	Forward Primer (5′ to 3′)	Reverse Primer (5′ to 3′)
Arginase	NC_000076.6	GGAACCCAGAGAGAGCATGA	TTTTTCCAGCAGACCAGCTT
Caspase 3	NC_000074.6	GGGCCTGTTGAACTGAAAAA	CCGTCCTTTGAATTTCTCCA
CD68	NC_000077.6	GGCCAAGCTATTGCGACATG	CCGAACACAGCGTAGATAGAC
CD163	NC_000072.6	GGGTCATTCAGAGGCACACTG	CTGGCTGTCCTGTCAAGGCT
F4/80	NC_000083.6	AAGCATCCGAGACACACACA	GGCAAGACATACCAGGGAGA
GAPDH	NC_000072.6	GTGTTCCTACCCCCAATGTA	AGGAGACAACCTGGTCCTCA
Grp78	NC_000068.7	TTCCTGCGTCGGTGTATTCA	GCGGTTGCCCTGATCGT
IL-1β	NC_000068.7	GGGCCTCAAAGGAAAGAATC	TACCAGTTGGGGAACTCTGC
iNOS	NC_000077.6	CCCCAAAGGGATGAGAAGTT	GGTCTGGGCCATAGAACTGA
MCP-1	NC_000077.6	CCCAATGAGTAGGCTGGAGA	TCTGGACCCATTCCTTCTTG
MPO	NC_000077.6	AACATGCAGCGCAGCCGG	AGCCCACAAAAGCGTCTC
TGF-β1	NC_000073.6	TGGAGCAACATGTGGAACTC	GTCAGCAGCCGGTTACCA
TNFα	NC_000083.6	CCCCAAAGGGATGAGAAGTT	GGTCTGGGCCATAGAACTGA

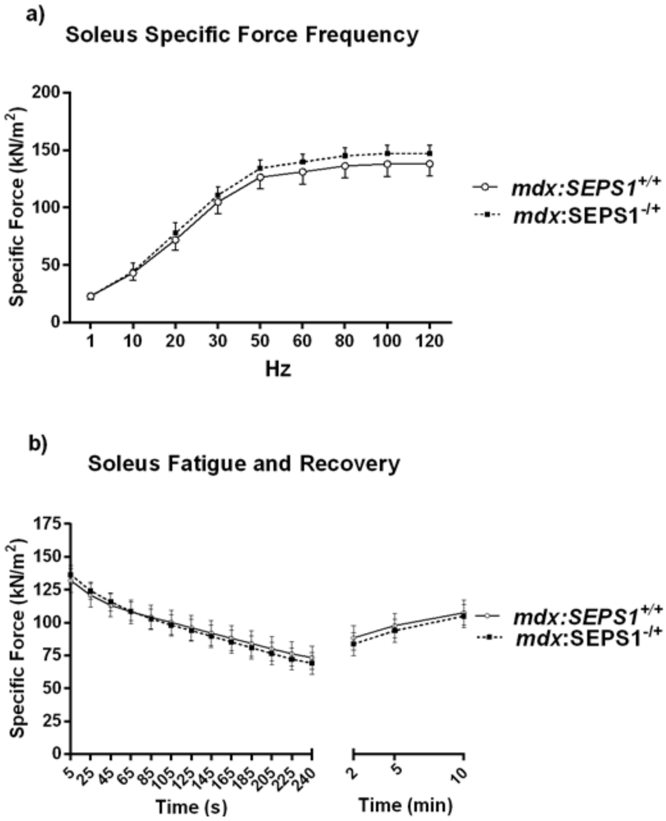

SUPPLEMENTARY FIGURE 1: Ex *vivo* strength and fatigue analysis of the soleus. **(a)** Force Frequency curve of the soleus muscle in response to *ex vivo* stimulation, and **(b)** specific force production of the soleus during a 4 minute submaximal fatiguing stimulation, and force recovery at 2, 5 and 10 minutes (n=11).

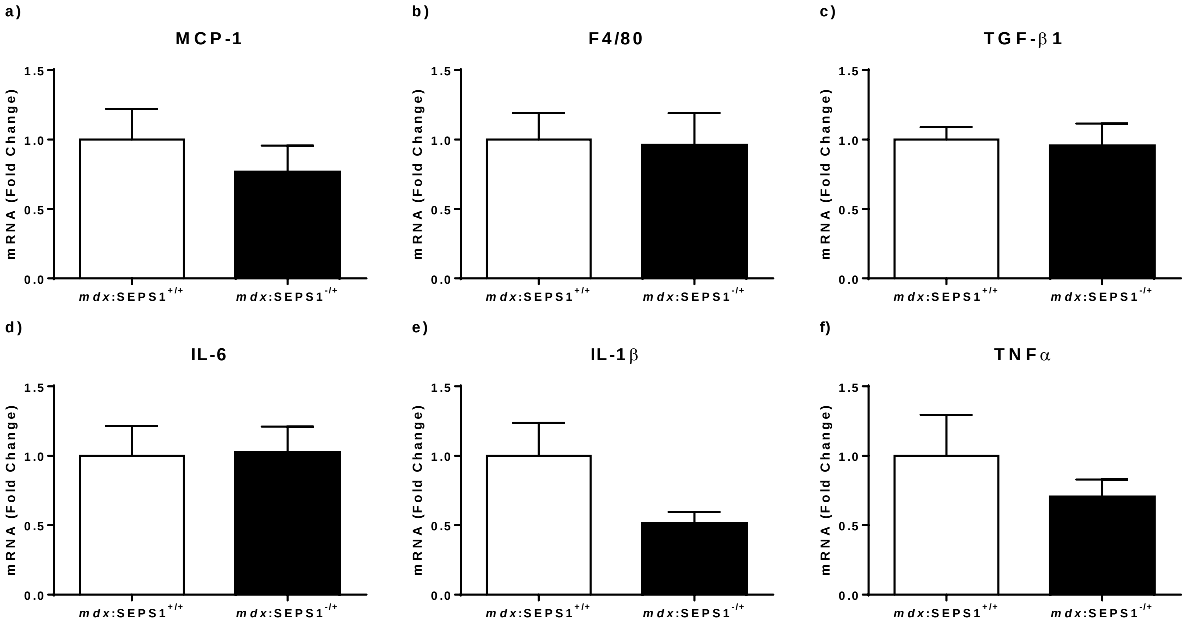

SUPPLEMENTARY FIGURE 2: Inflammatory gene profile of the soleus. (a) Monocyte chemoattractant protein 1 (MCP-1), **(b)** EGF-like module-containing mucin-like hormone receptor-like 1 (F4/80), **(c)** transforming growth factor β1 (TGF-β1), **(d)** interleukin 6 (IL-6), **(e)** interleukin 1β (IL-1β) and **(f)** tumour necrosis factor α (TNFα) gene expression in the soleus muscle at 12 weeks of age. Data are represented as fold change ± SEM, and are normalised to GAPDH. No differences in GAPDH expression were present between groups (n=11).
