# Supplementary material for: A Reduction in Selenoprotein S Amplifies the Inflammatory Profile of Fast-Twitch Skeletal Muscle in the mdx Dystrophic Mouse
Source: Mediators Inflamm. 2017 May 16;2017:7043429. doi: 10.1155/2017/7043429 (PMC5448157; doi:10.1155/2017/7043429)
Supplement: Supplementary file 2 [file 7043429.f2.pdf]

Supplementary Table 1: Mouse Primers Used for qPCR. *Cluster of differentiation 68 (Cd68), cluster of differentiation 163 (Cd163), EGF-like module-containing mucin-like hormone receptor-like 1 (F4/80), glyceraldehyde 3-phosphate dehydrogenase (Gapdh), glucose-regulated protein 78 (Grp78), interleukin 1β (Il-1β), inducible nitric oxide (iNos), monocyte chemoattractant protein 1 (Mcp-1), myeloperoxidase (Mpo), transforming growth factor β1 (Tgf-β1),tumour necrosis factor α (Tnfα).*

Gene	GenBank Accession	Forward Primer (5' to 3')	Reverse Primer (5' to 3')
Arginase	NC_000076.6	GGAACCCAGAGAGAGCATGA	TTTTTCCAGCAGACCAGCTT
Caspase 3	NC_000074.6	GGGCCTGTTGAACTGAAAAA	CCGTCCTTTGAATTTCTCCA
Cd68	NC_000077.6	GGCCAAGCTATTGCGACATG	CCGAACACAGCGTAGATAGAC
Cd163	NC_000072.6	GGGTCATTCAGAGGCACACTG	CTGGCTGTCCTGTCAAGGCT
F4/80	NC_000083.6	AAGCATCCGAGACACACACA	GGCAAGACATACCAGGGAGA
Gapdh	NC_000072.6	GTGTTCCTACCCCCAATGTA	AGGAGACAACCTGGTCCTCA
Grp78	NC_000068.7	TTCCTGCGTCGGTGTATTCA	GCGGTTGCCCTGATCGT
Il-1β	NC_000068.7	GGGCCTCAAAGGAAAGAATC	TACCAGTTGGGGAACTCTGC
iNos	NC_000077.6	CCCCAAAGGGATGAGAAGTT	GGTCTGGGCCATAGAACTGA
Mcp-1	NC_000077.6	CCCAATGAGTAGGCTGGAGA	TCTGGACCCATTCCTTCTTG
Mpo	NC_000077.6	AACATGCAGCGCAGCCGG	AGCCCACAAAAGCGTCTC
Tgf-β1	NC_000073.6	TGGAGCAACATGTGGAACTC	GTCAGCAGCCGGTTACCA
Tnfα	NC_000083.6	CCCCAAAGGGATGAGAAGTT	GGTCTGGGCCATAGAACTGA
