# Supplementary material for: A Reduction in Selenoprotein S Amplifies the Inflammatory Profile of Fast-Twitch Skeletal Muscle in the mdx Dystrophic Mouse
Source: Mediators Inflamm. 2017 May 16;2017:7043429. doi: 10.1155/2017/7043429 (PMC5448157; doi:10.1155/2017/7043429)
Supplement: Supplementary file 3 [file 7043429.f3.pdf]

a)

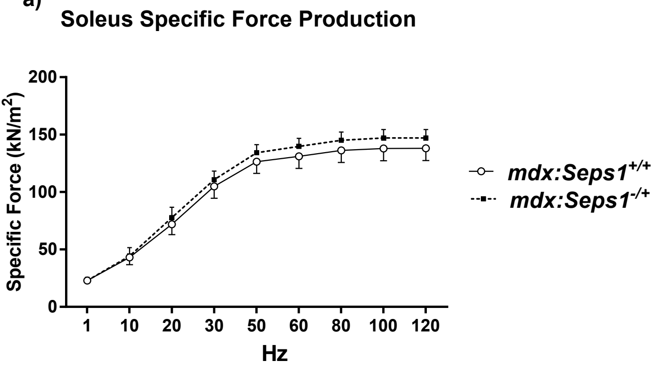

b)

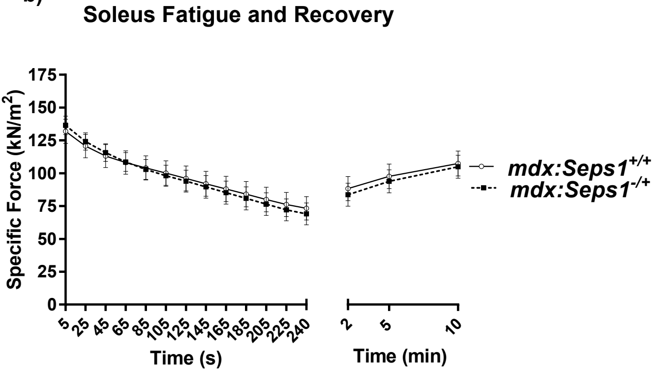

Supplementary Figure 1: Ex vivo strength and fatigue analysis of the soleus. (a) Force Frequency curve of the soleus muscle in response to ex vivo stimulation, and (b) specific force production of the soleus during a four minute submaximal fatiguing stimulation, and force recovery at 2, 5 and 10 minutes (n=11).
