# Supplementary material for: A Reduction in Selenoprotein S Amplifies the Inflammatory Profile of Fast-Twitch Skeletal Muscle in the mdx Dystrophic Mouse
Source: Mediators Inflamm. 2017 May 16;2017:7043429. doi: 10.1155/2017/7043429 (PMC5448157; doi:10.1155/2017/7043429)
Supplement: Supplementary file 4 [file 7043429.f4.pdf]

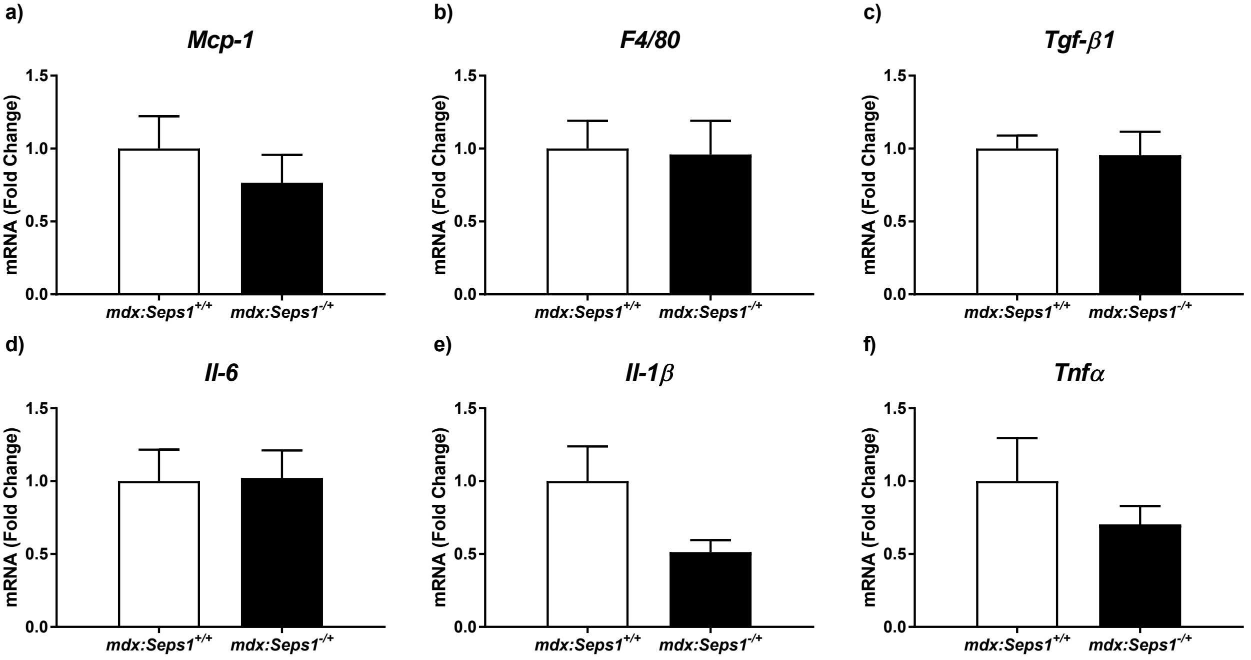

Supplementary Figure 2: Inflammatory gene profile of the soleus. (a) Monocyte chemoattractant protein 1 (Mcp-1), (b) EGF-like module-containing mucin-like hormone receptor-like 1 (F4/80), (c) transforming growth factor β1 (Tgf-β1), (d) interleukin 6 (Il-6), (e) interleukin 1β (Il-1β) and (f) tumour necrosis factor α (Tnfα) gene expression in the soleus muscle at 12 weeks of age. Data are represented as fold change ± SEM, and are normalised to Gapdh. No differences in Gapdh expression were present between groups (n=11).
